# Comparative Neuroprotective Effects of Dexamethasone and Minocycline during Hepatic Encephalopathy

**DOI:** 10.1155/2014/254683

**Published:** 2014-02-17

**Authors:** Maha Gamal, Zainab Abdel Wahab, Mohamed Eshra, Laila Rashed, Nivin Sharawy

**Affiliations:** ^1^Department of Physiology, Faculty of Medicine, Cairo University, Giza 11562, Egypt; ^2^Department of Biochemistry, Faculty of Medicine, Cairo University, Giza 11562, Egypt; ^3^Department of Anesthesia, Pain Management and Perioperative Medicine, Faculty of Medicine, Dalhousie University, 5850 College Street, Halifax, NS, Canada B3H 2Y9

## Abstract

*Objective.* Encephalopathy and brain edema are serious complications of acute liver injury and may lead to rapid death of patients. The present study was designed to investigate the role of the inflammatory mediators and oxidative stress in the cytotoxic brain oedema and the neuroprotective effects of both minocycline and dexamethasone. *Methods.* 48 male albino rats were divided into 4 groups: control group, acute liver injury (ALI) group, minocycline pretreated ALI group, and dexamethasone pretreated ALI group. 24 hours after acute liver injury serum ammonia, liver enzymes, brain levels of heme oxygenase-1 gene, iNOS gene expression, nitrite/nitrate, and cytokines were measured. In addition, the grades of encephalopathy and brain water content were assessed. *Results.* ALI was associated with significant increases in all measured inflammatory mediators, oxidative stress, iNOS gene expression, and nitrite/nitrate. Both minocycline and dexamethasone significantly modulated the inflammatory changes and the oxidative/nitrosative stress associated with ALI. However, only minocycline but not dexamethasone significantly reduced the cytotoxic brain oedema. *Conclusion.* Both minocycline and dexamethasone could modulate inflammatory and oxidative changes observed in brain after ALI and could be novel preventative therapy for hepatic encephalopathy episodes.

## 1. Introduction

Intracranial hypertension is a major cause of morbidity and mortality of patients suffering from acute liver injury (ALI). Cytotoxic brain oedema, the increase in cerebral blood volume and the increase in cerebral blood flow, in part due to inflammation, to glutamine, and to toxic products of the diseased liver, are major factors contributing to intracranial hypertension [1].

Patients, who have experienced hepatic encephalopathy, are at risk for recurrence. Preventative maintenance therapy is recommended to reduce the risk of repeat episodes and hospitalization and will have a great economic, social, familial, and personal implication [2].

Certain antibiotics, in addition to their anti-infectious qualities, have immunomodulatory properties [2]. Minocycline, owing to its relatively small size and highly lipophilic nature, can cross the blood-brain barrier (BBB) with ease and has been shown to penetrate the cerebrospinal fluid (CSF) [3]. In addition, minocycline inhibits the microglial activation and the proinflammatory cytokines production, delays the progression of encephalopathy, and attenuates brain oedema [4]. Dexamethasone is the most commonly used corticosteroid. Recent studies have shown that corticosteroids reduce oedema through decreasing the capillary permeability and the expression of vascular endothelium growth factor (VEGF) associated with brain tumours [5, 6]. In addition, the inhibition of nuclear factor kappa-light-chain-enhancer of activated B cells (NF-*κ*B) activation can account for many of the anti-inflammatory properties of dexamethasone [7].

The present study is designated to investigate the possible role of oxidative/nitrosativestress and inflammatory changes in the brain oedema induced by acute liver injury and the protective effects of minocycline and dexamethasone.

## 2. Material and Methods

### 2.1. Animals

After obtaining approval from the institutional Animal Care Committee, we used 48 male albino rats (body weight: 200–250 g, Laboratory Animal House Unit of Faculty of Medicine, Cairo University). The animals were housed in chip-bedded cages and, prior to experiments, acclimated for 1 week in the air-conditioned institutional animal care unit. They were housed under 12-hour light/dark cycles, with free access to water and standard rat chow.

### 2.2. General Protocol

Male rats were randomly assigned in to one of the 4 groups. Each group was divided into 2 subgroups: subgroup A, in which rats were used for assessment of brain and blood parameters, and subgroup B for the assessment of the degree of encephalopathy after injection of galactosamine for 7 days (6 rats in each subgroup). The main 4 groups included the control placebo treated group, the acute hepatic injury group, and the two pretreated groups, in which either minocycline (Sigma-Aldrich, Spruce St., St Louis, USA) (22.5 mg/kg twice daily; i.p.) or dexamethasone (Sigma-Aldrich, Spruce St., St Louis, USA) (1 mg/kg daily) was injected intraperitoneally 2 days before induction of acute hepatic injury.

Acute hepatic injury was induced by the highly specific hepatotoxin galactosamine (Sigma-Aldrich, Spruce St., St Louis, USA) (2.5 g/kg i.p.) [8]. All animals were sacrificed 24 h after galactosamine injection. The blood and brain samples were used for further determination of alanine transaminase (ALT), aspartate transaminase (AST), ammonia, levels of interleukins (IL-1*β*, IL-6), TNF-*α*, heme oxygenase-1 gene expression, inducible nitric oxide synthase (iNOS) gene expression, nitrite/nitrate levels, and the brain oedema.

Measuring the absolute water content of the brain was carried out using the wet-weight/dry-weight method. The brain was weighed before and after 24-hour incubation in a 100°C oven. Water content of the brain samples was expressed as percentage of water content according to the following equation: % Water = (Wet Weight − Dry Weight)/Wet Weight × 100 [9].

Animals were assessed neurologically after injection of galactosamine for seven days and during the progression of acute liver injury. The grades of hepatic encephalopathy were assigned as follows: Grade 0: normal motor activity (voluntary movement); Grade I: decreased motor activity and sedation, but righting reflex is present; Grade II: no righting reflex, and when the rat is put on its back, it turns over immediately; Grade III: no reaction to pain stimuli and no corneal reflex [10].

### 2.3. Serum ALT and AST

The serum alanine aminotransferase (ALT) and aspartate aminotransferase (AST) were detected by colorimetric method. Each blood sample was placed in dry clean centrifuge tube and then centrifuged for 10 minutes at 3000 round per minute (rpm) to separate the serum. Serum was carefully separated into clean dry Wassermann tubes by using a Pasteur pipette and kept frozen at −70°C until analysis by commercial available kit (Sigma-Aldrich, Spruce St., St Louis, USA).

### 2.4. Serum Ammonia

Ammonia was enzymatically determined in serum using Ammonia Assay Kit (Sigma-Aldrich, Spruce St., St Louis, USA). Ammonia reacts with *α*-ketoglutaric acid (KGA) and reduced nicotinamide adenine dinucleotide phosphate (NADPH) in the presence of L-glutamate dehydrogenase (GDH) to form L-glutamate and oxidized nicotinamide adenine dinucleotide phosphate (NADP+). The decrease in absorbance at 340 nm, due to the oxidation of NADPH, is proportional to the ammonia concentration. L-Glutamate dehydrogenase reacts specifically with ammonia. The Ammonia Assay Kit may be used to determine ammonia concentrations in the range of 0.2–15 *μ*g/mL.

### 2.5. Real-Time Polymerase Chain Reaction (Real-Time PCR)

Real-time PCR (Pomega, Madison, WI, USA) was used to measure iNOS and heme oxygenase-1 gene expression. The key equipment for real-time PCR is a specialized thermocycler with fluorescence detection modules, which is used to monitor and record the fluorescence in real time as amplification occurs. A typical workflow of real-time PCR for gene expression measurement involves RNA isolation, reverse transcription, real-time PCR assay development, real-time PCR experiment, and data analysis.

### 2.6. Determination of IL-1*β*, IL-6, TNF-*α*, and IL-10 Levels in Brain

The concentration of cytokines was measured by the sandwich Enzyme Linked Immuno-Sorbent Assay (ELISA) kits (Thermo scientific, Meridian road, Rockford, USA; Invitrogen Corporation, Flynn Road, Camarillo, CA;R&D Systems, McKinley Place NE, Minneapolis, USA).

### 2.7. Griess Reaction Assay

Nitrite concentration in the brain tissue was determined by a colorimetric assay based on the Griess Reaction (AMRESCO Inc., Cochran Rd., USA). Nitrite is converted to nitrous acid which then diazotizes sulfanilamide. The sulfanilamide-diazonium salt then reacts with N-(1-Naphthyl)-ethylenediamine (NED) to form an intense purple-colored diazo compound. Its concentration is readily determined by measuring the absorbance at 540/550 nm. The absorbance of this adduct at 540 nm is linearly proportional to the nitrite concentration in the sample.

### 2.8. Statistical Methods

The results are given as means ± standard deviation (SD). Results were analyzed by using the software Prism 5 (GraphPad Software, La Jolla, CA, USA). Comparisons between groups were done using analysis of variance (ANOVA) with multiple comparisons post hoc test in normally distributed quantitative variables. For comparing categorical data, the Chi-square (*χ* ^2^) test was performed. *P* values less than 0.05 were considered as statistically significant.

## 3. Results

### 3.1. Oxidative/Nitrosative Stress and the Inflammatory Changes Associated with Brain Oedema

**Figure 1:**
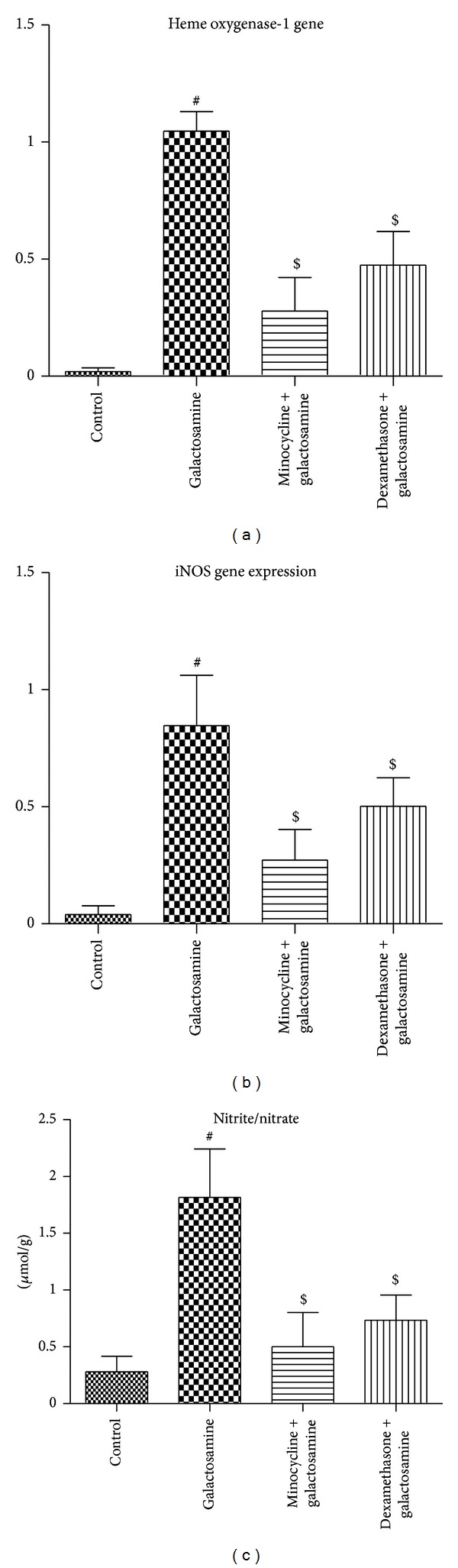
The effects of minocycline and dexamethasone on the oxidative/nitrosative stress associated with minimal hepatic encephalopathy. Both dexamethasone and minocycline (*n* = 6 per group) reduced the levels of heme oxygenase-1 gene expression (a), iNOS gene expression (b), and nitrite/nitrate (*μ*mol/g) (c) in the brain tissue associated with acute liver injury. All values are expressed in means ± SD. ^#^ *P* < 0.05 versus control; ^$^ *P* < 0.05 versus galactosamine treated group.

**Figure 2:**
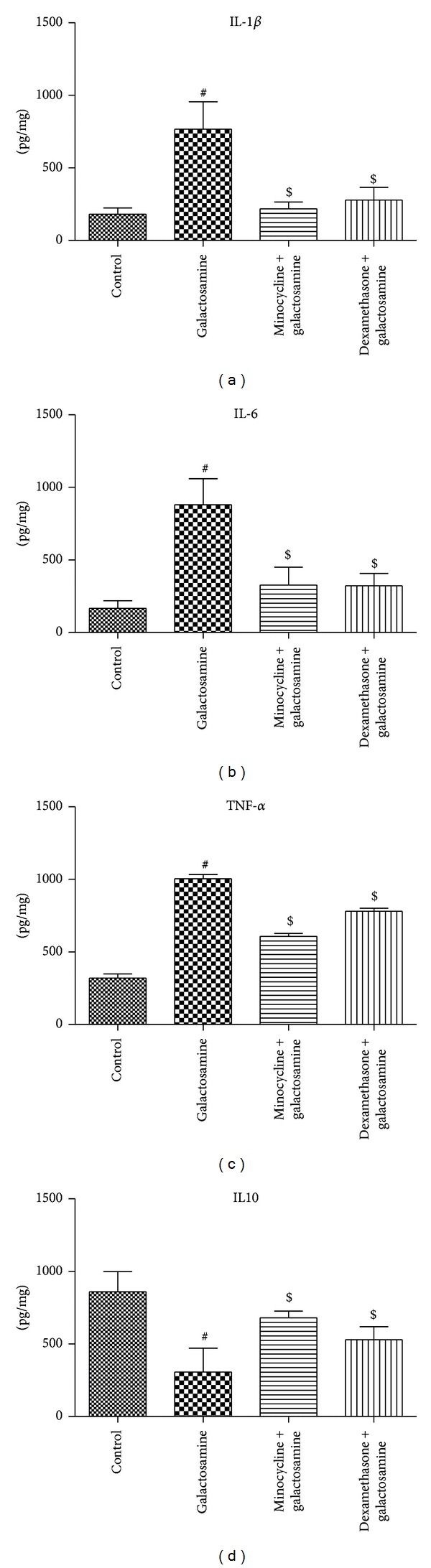
The effects of minocycline and dexamethasone on the inflammatory changes associated with minimal hepatic encephalopathy. Both dexamethasone and minocycline (*n* = 6 per group) reduced the levels of IL-1*β* (pg/mg) (a), IL-6 (pg/mg) (b), and TNF-*α* (pg/mg) (c) and increased the level of IL-10 (pg/mg) (d) in the brain tissue associated with acute liver injury. All values are expressed in means ± SD. ^#^ *P* < 0.05 versus control; ^$^ *P* < 0.05 versus galactosamine treated group.

**Figure 3:**
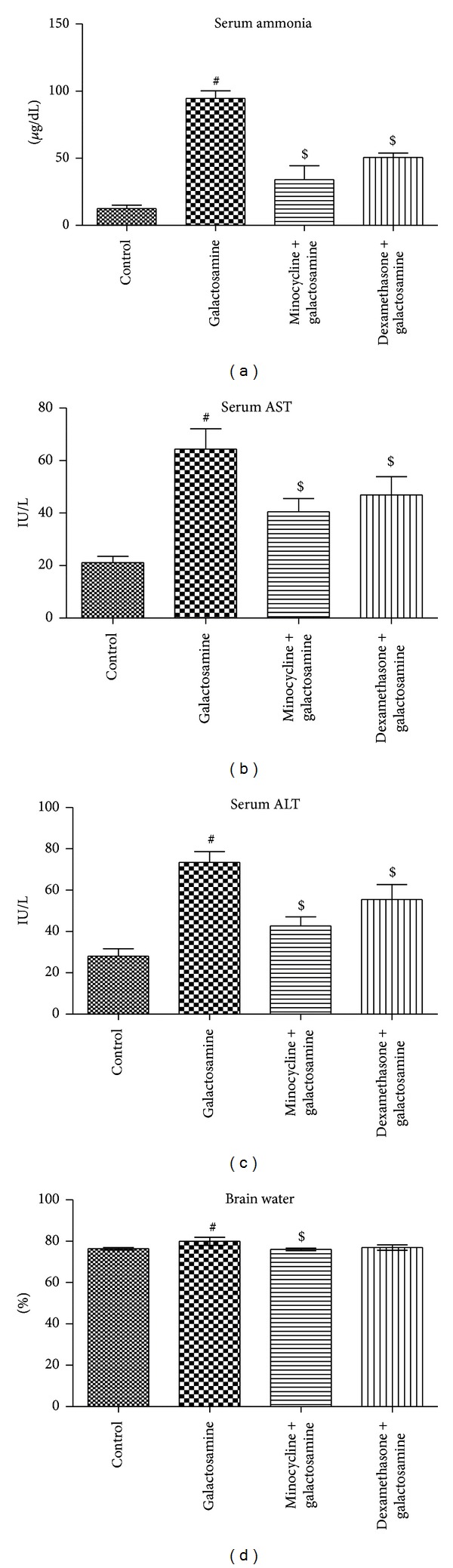
The effects of minocycline and dexamethasone on the acute liver injury and brain oedema. Both dexamethasone and minocycline (*n* = 6 per group) reduced the levels of serum ammonia (*μ*g/dL) (a), AST (IU/L) (b), ALT (IU/L) (c), and the brain water (%) (d) associated with acute liver injury. All values are expressed in means ± SD. ^#^ *P* < 0.05 versus control; ^$^ *P* < 0.05 versus galactosamine treated group.

We found significant increases (*P* < 0.05) in liver enzymes (ALT (IU/L) 28.07 ± 3.61 versus 73.47 ± 5.25; AST (IU/L) 21.13 ± 2.35 versus 64.38 ± 7.71), ammonia (*μ*g/dL) (12.55 ± 2.48 versus 94.58 ± 5.58), heme oxygenase-1 gene expression (0.02 ± 0.02 versus 1.05 ± 0.08), iNOS gene expression (0.04 ± 0.04 versus 0.85 ± 0.22), nitrite/nitrate (*μ*mol/g) (0.28 ± 0.13 versus 1.82 ± 0.43), IL-1 (Pg/mg) (181.03 ± 42.86 versus 766.95 ± 190.18), IL-6 (Pg/mg) (166.87 ± 52.39 versus 880.93 ± 178.73), TNF (Pg/mg) (319.07 ± 70.89 versus 1004.87 ± 72.32), and brain water (%) (76.34 ± 0.59 versus 79.97 ± 1.92) and a significant decrease (*P* < 0.05) in IL-10 (Pg/mg) (859.65 ± 138.22 versus 306.62 ± 164.41) in the brain tissue after acute liver injury induced by galactosamine in comparison to the control group (Figures 1, 2, and 3).

We observed also decreasing in the motor activity and sedation in 50% of rats two days after galactosamine induced ALI. In addition, all rats with ALI (nonpretreated and pretreated) showed high mortality (16.6%) on the first day and (83.3%) 2 days after galactosamine injection.

### 3.2. Anti-Inflammatory/Antioxidant Effects of the Minocycline

We found that pretreatment of rats with minocycline was associated with significant decreases (*P* < 0.05) in the liver enzymes (ALT (IU/L) 42.7 ± 4.4; AST (IU/L) 40.48 ± 5.1), ammonia (*μ*g/dL) (34.07 ± 10.32), heme oxygenase-1 gene expression (0.28 ± 0.14), iNOS gene expression (0.27 ± 0.13), nitrite/nitrate (*μ*mol/L) (0.5 ± 0.3), IL-1 (Pg/mg) (217.75 ± 48.41), IL-6 (Pg/mg) (326.53 ± 123.14), TNF-*α* (Pg/mg) (606.33 ± 53.43), and brain water (%) (76.03 ± 0.57) and a significant increase (*P* < 0.05) in IL-10 (Pg/mg) (680.15 ± 46.57) associated with acute liver injury, Figures 1–3. In addition, four rats (66.7%) showed no impairment of motor activity but with no significant difference in comparison to nonpretreated ALI group (33.3%). However, no significant difference was observed in mortality rate (0%) on the first day and (100%) on the second day.

### 3.3. Antioxidant Anti-Inflammatory Effects of Dexamethasone

Pretreatment of rats with dexamethasone was associated with significant reduction (*P* < 0.05) in the levels of the liver enzymes (ALT (IU/L) 55.48 ± 7.19; AST (IU/L) 46.9 ± 7), ammonia (*μ*g/dL) (50.52 ± 3.28), oxidative stress (0.48 ± 0.14), iNOS gene expression (0.5 ± 0.12), nitrite/nitrate (*μ*mol/g) (0.73 ± 0.22), IL-1 (Pg/mg) (278.2 ± 87.51), IL-6 (Pg/mg) (321.38 ± 86.03), TNF (Pg/mg) (781.2 ± 47.48), but not brain water (%) (76.95 ± 1.36), which were increased after acute liver injury. In addition, IL-10 (Pg/mg) (528.77 ± 90.97) was significantly increased (*P* < 0.05) in dexamethasone pretreated group (Figures 1–3).

In addition, four rats (66.7%) showed no impairment of motor activity, but not significant in comparison to nonpretreated ALI group (33.3%), and no significant difference was observed in mortality rate (0%) on the first day and (100%) on the second day.

## 4. Discussion

Hepatic encephalopathy and brain edema are serious central nervous system complications of liver failure, in which rapid deterioration of liver function results in altered mentation and coagulation, and associated commonly with drug induced liver injury, viral hepatitis, autoimmune liver disease, and shock [11, 12]. Cerebral edema may contribute to ischemic and hypoxic brain injury, which may result in long-term neurological deficits in survivors.

For the first time, in our comparative study, minocycline was found to be more effective, in the reduction of brain oedema associated with galactosamine induced acute liver injury, than dexamethasone. However, no significant differences in neuroprotective anti-inflammatory effects of both dexamethasone and minocycline were observed.

Ammonia is directly toxic to the brain [13]. In our study, we found that high level of ammonia, which maybe contributed to insufficient detoxification by the liver and muscle and the decrease in urinary loss of ammonia due to alkalosis [13–16], is associated with high levels of IL-1*β*, IL-6, TNF-*α*, heme oxygenase-1 gene expression, iNOS gene expression, and nitrite/nitrate. In our result, we found also decreasing in motor activity and sedation two days after galactosamine injection; this could have contributed to the acute onset of liver insult, which has been intimately associated with the brain edema [17].

Our results suggest that ammonia induced oxidative/nitrosative stress and inflammatory changes could be the main determinant factor for brain oedema associated with hepatic encephalopathy. The mechanism through which free radical production is increased is currently not fully understood. Animal studies have shown significant reductions in the activities of glutathione peroxidase and superoxide dismutase enzymes, both in the liver and brain of rats exposed to ammonium acetate [18]. In addition, in vivo excessive ammonia is associated with N-methyl D-aspartate (NMDA) receptor activation, which reduces antioxidant enzyme activity and results in increased production of superoxide anions [19]. In addition, activation of NMDA receptors would lead to the increase of intracellular calcium and trigger the mitochondrial production of reactive oxygen species on one hand and would induce the transcription of the iNOS gene, following degradation of the calcium-dependent I*κ*B, an inhibitor of kappa B protein that inactivates the NF-*κ*B transcription, resulting in increased production of NO and peroxynitrite [20, 21].

Additional proposed mechanism includes ammonia induced activation of mitogen-activated protein kinases and activation of nuclear factor-*κ*b. All of which can lead to enhanced cytokines activity [22]. TNF-*α*, IL-1*β*, and IL-6, individually and in combination, induce astrocyte swelling [23]. NF-*κ*B is known also to activate various genes, including inducible nitric oxide synthase (iNOS) and NADPH oxidase, whose products: nitric oxide, superoxide, and peroxynitrite have been shown to cause astrocyte swelling [23]. The presence of oxidative/nitrosative stress can also influence the expression or the activity of enzymes involved in the regulation of blood flow and can induce the cerebrovascular vasodilatation through activation of potassium channels [24].

Our results further support the recent studies, which suggest the central anti-inflammatory role of minocycline in acute liver injury, through modulation of microglia, immune cell activation, and subsequent release of cytokines, chemokines, lipid mediators of inflammation and nitric oxide (NO) release [20, 21]. In addition, minocycline inhibits oxidative stress in astrocytes, through direct radical scavenging activity and enhancement of antioxidant defenses [21, 25, 26].

We found also that dexamethasone significantly reduced the heme-oxygenase-1 gene expression, iNOS gene expression, nitrite/nitrate, and the proinflammatory cytokines [IL-1*β*, IL-6, and TNF-*α*] and increased IL-10 associated with acute liver injury, with no significant differences in comparison to minocycline. The most general known effects of dexamethasone are to inhibit the synthesis, release, and/or efficacy of cytokines and other mediators that promote immune and inflammatory reactions [27]. This is through its inhibitory effects on nuclear factor *κβ* signaling pathways [28]. Our study further supports few studies, which showed the significant effects of corticosteroid in relieving oxidative stress [29–31]. The antioxidant effect of dexamethasone could be due to alteration in level of enzymatic and nonenzymatic antioxidant status, such as superoxide dismutase, catalase, and glutathione peroxidase—enzymes [32–35].

An interesting finding in our study is the ability of minocycline to reduce brain oedema, in spite of the fact that no differences in the anti-inflammatory properties of both minocycline and dexamethasone were observed. Canalese et al. [36] were unable also to show any benefit of dexamethasone in the reduction of brain oedema and suggested that glucocorticoids are more effective in oedema that is focal and chronic than the more diffuse and acute varieties, which are associated with acute liver injury [37]. Whether this observation contributed to direct effects of minocycline on astrocyte cells, cerebral blood flow regulation or the blood brain barrier permeability needs further investigations [38].

Another finding in our study was that minocycline induced significant reduction in AST, ALT, and ammonia. Josephs et al. [39] suggested that D-galactosamine, frequently used as a model of acute hepatic injury, is presumed to enhance macrophage/monocyte activation and that the secreted TNF-*α* and its binding to the TNFR-I are essential for the hepatic injury in this model. Since treatment with minocycline was reported to be associated with liver injury [40, 41], the anti-inflammatory effects of minocycline on the galactosamine induced liver injury, which could be due to TNF-*α*, need further investigation.

In our study, we observed that dexamethasone also reduced the ammonia and liver enzymes. This could be due to attenuation of the liver injury through downregulation of glucocorticoid induced tumor necrosis factor receptor ligand (GITRL) in Kupffer cells (KC) and inhibition of TNF-*α* and IL-6 expression [42]. However, this finding needs further investigation.

The study has limitations. Although galactosamine induced ALI was selected as an ideal model that fulfilled the following criteria: (1) potential reversibility of the lesion, (2) reproducibility of the clinical picture, and (3) minimal hazard to personnel unlike any other virus models [43], we found that the degree of ALI induced brain oedema was mild, and this could be due to high mortality rate, the short observation period, and the gradual development of encephalopathy in galactosamine induced ALI [44]. However, minimal hepatic encephalopathy is more widely recognized and may be precipitated by bleeding, infection, medications, or even dehydration and its prevention will dramatically improve the quality of life for many patients [40, 41]. In addition, one-time point was chosen according to previously published studies that investigated the anti-inflammatory effect of minocycline [25]. However, further investigation of different time points that may reduce also the mortality rate is needed. Finally, our findings need further confirmation by larger sample size experimental study.

## 5. Conclusion

Our findings demonstrate that acute liver injury could lead to an early inflammatory changes and activation of the oxidative stress, which could contribute to the cytotoxic brain oedema. On this basis, early prevention with minocycline or dexamethasone may yield favorable protective results on the brain pathophysiological parameters which are adversely affected due to acute liver injury. In spite of the fact that both minocycline and dexamethasone have anti-inflammatory properties, only minocycline was effective in reducing brain oedema. This observation needs further investigation.
